# deepBase v3.0: expression atlas and interactive analysis of ncRNAs from thousands of deep-sequencing data

**DOI:** 10.1093/nar/gkaa1039

**Published:** 2020-11-11

**Authors:** Fangzhou Xie, Shurong Liu, Junhao Wang, Jiajia Xuan, Xiaoqin Zhang, Lianghu Qu, Lingling Zheng, Jianhua Yang

**Affiliations:** MOE Key Laboratory of Gene Function and Regulation, State Key Laboratory for Biocontrol, The Fifth Affiliated Hospital, Sun Yat-sen University, Guangzhou 510275, China; MOE Key Laboratory of Gene Function and Regulation, State Key Laboratory for Biocontrol, The Fifth Affiliated Hospital, Sun Yat-sen University, Guangzhou 510275, China; MOE Key Laboratory of Gene Function and Regulation, State Key Laboratory for Biocontrol, The Fifth Affiliated Hospital, Sun Yat-sen University, Guangzhou 510275, China; MOE Key Laboratory of Gene Function and Regulation, State Key Laboratory for Biocontrol, The Fifth Affiliated Hospital, Sun Yat-sen University, Guangzhou 510275, China; School of Medicine, South China University of Technology, Guangzhou 510275, China; MOE Key Laboratory of Gene Function and Regulation, State Key Laboratory for Biocontrol, The Fifth Affiliated Hospital, Sun Yat-sen University, Guangzhou 510275, China; MOE Key Laboratory of Gene Function and Regulation, State Key Laboratory for Biocontrol, The Fifth Affiliated Hospital, Sun Yat-sen University, Guangzhou 510275, China; MOE Key Laboratory of Gene Function and Regulation, State Key Laboratory for Biocontrol, The Fifth Affiliated Hospital, Sun Yat-sen University, Guangzhou 510275, China; Department of Interventional Medicine, The Fifth Affiliated Hospital, Sun Yat-sen University, Zhuhai 519000, China

## Abstract

Eukaryotic genomes encode thousands of small and large non-coding RNAs (ncRNAs). However, the expression, functions and evolution of these ncRNAs are still largely unknown. In this study, we have updated deepBase to version 3.0 (deepBase v3.0, http://rna.sysu.edu.cn/deepbase3/index.html), an increasingly popular and openly licensed resource that facilitates integrative and interactive display and analysis of the expression, evolution, and functions of various ncRNAs by deeply mining thousands of high-throughput sequencing data from tissue, tumor and exosome samples. We updated deepBase v3.0 to provide the most comprehensive expression atlas of small RNAs and lncRNAs by integrating ∼67 620 data from 80 normal tissues and ∼50 cancer tissues. The extracellular patterns of various ncRNAs were profiled to explore their applications for discovery of noninvasive biomarkers. Moreover, we constructed survival maps of tRNA-derived RNA Fragments (tRFs), miRNAs, snoRNAs and lncRNAs by analyzing >45 000 cancer sample data and corresponding clinical information. We also developed interactive webs to analyze the differential expression and biological functions of various ncRNAs in ∼50 types of cancers. This update is expected to provide a variety of new modules and graphic visualizations to facilitate analyses and explorations of the functions and mechanisms of various types of ncRNAs.

## INTRODUCTION

The vast majority of human genome and other mammalian genomes are transcribed to encode thousands of short (sRNAs) and long non-coding RNAs (lncRNAs), which have been implicated in diverse physiological and pathological processes, such as tumorigenesis, development, imprinting, apoptosis and cell differentiation (1–5). Although thousands of ncRNA studies have been published in recent years, only a small fraction of ncRNAs have been well functionally studied.

Given the thousands of ncRNAs being discovered in various species, many databases have been developed to help researchers understand their diversity and functions in recent years. Notable examples include miRBase (6), a reference database of published miRNA sequences and GENCODE (7), a reference database for lncRNAs. In addition, a series of databases have been developed to explore ncRNA expression patterns, regulatory networks and biological functions, such as RNAcentral (8), LNCipedia (9), LncRNAdb (10), ChIPBase (11), NONCODE (12), LncRNADisease (13), starBase (14) and circBase (15). However, these databases focus on either specific ncRNA families or specific features of ncRNAs.

Tremendous amounts of deep-sequencing data have been generated by multiple consortium projects, such as the ENCODE (16), TCGA (17), ICGC (18), GTEx (19) and ERCC (20) projects, providing new opportunities to understand the functions of ncRNAs. A few databases have integrated TCGA RNA-seq data to explore the expression profiles of miRNAs and lncRNAs in cancers. Notable examples include starBase (14), which enables the pan-cancer analysis on miRNA-target and RBP-RNA interactions in ∼10 000 clinical samples of 32 types of cancers, and TANRIC (21) which is an interactive open platform for exploration of the functions of lncRNAs in cancer. However, these databases use only one consortium project (e.g. TCGA) to explore two types of ncRNAs (e.g. miRNAs and lncRNAs). There is a great need to integrate all deep-sequencing data produced by all large consortium projects to explore the dynamic expression, clinical implications and functions of various ncRNAs in physiological and pathological processes.

**Figure 1.**
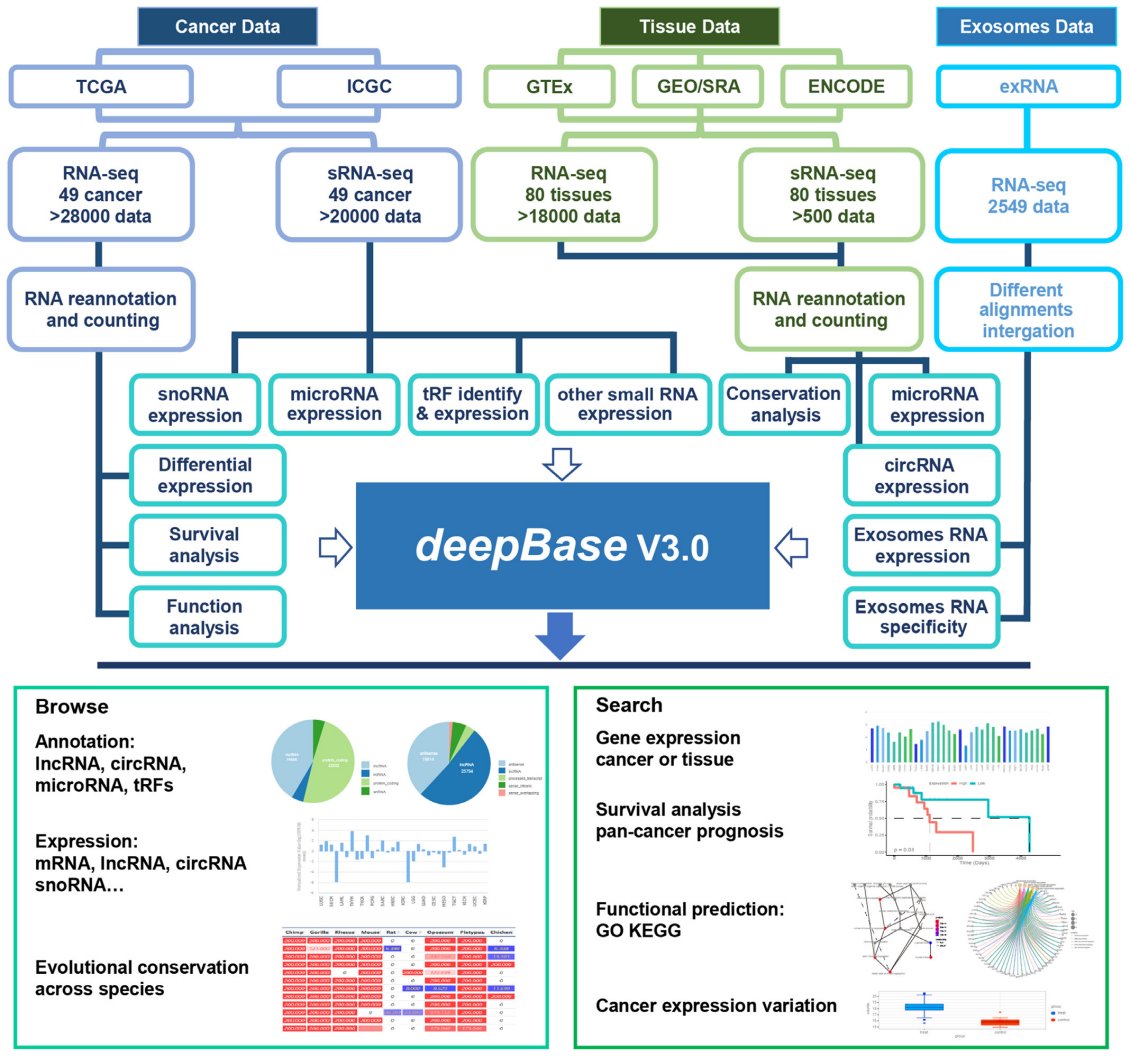
System-level overview of the deepBase v3.0 framework. We performed a large-scale integration and analysis of ∼46 000 RNA-seq datasets and 20 000 sRNA-seq datasets downloaded from large consortium projects and various databases. All small and large ncRNAs were identified and annotated. We constructed the most comprehensive database of expression, evolution, prognosis and extracellular patterns and functional predictions for various types of ncRNAs. All results are stored in MySQL relational databases and displayed in interactive webpages.

To overcome the abovementioned issues, we have updated deepBase (22) to version 3.0 (deepBase v3.0, Figure 1, Table 1). deepBase v3.0, for the first time, constructs the expression profiles of tRFs and snoRNAs by mining small RNA deep-sequencing data from TCGA. deepBase v3.0 also provides the most comprehensive expression profiles available for lncRNAs and other ncRNAs in normal and cancer tissues by integrating sequencing data from large consortium projects, including the ENCODE, TCGA, ICGC and GTEx projects. By analyzing sequencing data from the ERCC, we decoded the extracellular patterns of miRNAs, lncRNAs and circRNAs. In addition, deepBase v3.0 provides a variety of new web modules and graphic visualizations to facilitate analyses and explorations of the complex expression, functions and evolution of various types of ncRNAs.

**Table 1.** Major improvements of deepBase v3.0. This table describes the major improvements of data features and web-based functionalities in the updated deepBase v3.0 compared to previous versions

Data features/functionalities	deepBase v2.0	deepBase v3.0
Total RNA-seq data	558	46 384
Total sRNA-seq data	478	21 235
Tissue RNA samples	489	18 685
LncRNA information entries	367 539	436 436
Cancer RNA profiling	none	49 cancer types
Cancer RNA-seq data	none	25 639
Cancer sRNA-seq data	none	20 707
Patients clinical samples	none	14 810
Cancer mRNA expression	none	19 814 genes
Cancer lncRNA expression	none	14 855 genes
Cancer sRNA expression	none	7495 genes
Cancer miRNA expression	none	1881 genes
Cancer snoRNA expression	none	1988 genes
tRFs annotation	none	>1100
tRFs expression and variation	none	31 cancer types
RNA differential expression	none	24 cancer types
Clinical prognosis	none	12 310 genes
Exosome RNA profiling	none	9 biofluids types
Exosome RNA-seq data	none	2549
Exosome mRNA expression	none	81 692 entries
Exosome lncRNA expression	none	81 229 entries
Exosome miRNA expression	none	3396 entries
Exosome circRNA expression	none	918 entries

## MATERIALS AND METHODS

### Integration of small RNA-seq and RNA-seq data

More than 28 000 RNA-seq and small RNA-seq (sRNA-seq) data from 14 species were collected from the GEO (23), GTEx (19), ENCODE (16), exRNA (20), TCGA (17) and ICGC (18). Datasets of normal tissue were retrieved from GTEx and ENCODE, and cancer-related datasets and the corresponding clinical data were downloaded from TCGA and ICGC. The human (*Homo sapiens*) reference genome was updated to GRCh38(NCBI GRCh38) (24). The genome sequences of other species, including the mouse (UCSC mm10), chicken (*Gallus gallus*, v4), chimp (*Pan troglodytes*, panTro4), gorilla (*Gorilla gorilla gorilla*, gorGor3), rhesus monkey (*Macaca mulatta*, rheMac3), rat (*Rattus norvegicus*, rn6), cow (*Bos taurus*, bosTau7), opossum (*Monodelphis domestica*, monDom5), platypus (*Ornithorhynchus anatinus*, ornAna1), *X. tropicalis* (*Xenopus tropicalis*, xenTro3), zebrafish (*Danio rerio*, danRer7) and *C. elegans* (*Caenorhabditis elegans*, ce10), were downloaded from the UCSC Genome Browser. Gene annotation files were downloaded from the UCSC Genome Browser corresponding to the genome versions (Table 2). For exosomes RNA, data for 2549 healthy samples were retrieved from exRNA database (20). Once collected, the data were classified into different species, tissue, cancer or exosome types according to the metafile descriptions or related literature.

**Table 2.** The genome and annotation sources of 13 species. This table describes the annotation version of genome used in the integration of sRNA-seq and RNA-seq data

Species	Assembly	Annotation Source
*Homo sapiens*	GRCh38(hg38)	UCSC Genome Browser
*Mus musculus*	GRCm38(mm10)	UCSC Genome Browser
*Gallus gallus*	galGal4	UCSC Genome Browser
*Pan troglodytes*	panTro4	UCSC Genome Browser
*Gorilla gorilla gorilla*	gorGor3	UCSC Genome Browser
*Macaca mulatta*	rheMac3	UCSC Genome Browser
*Rattus norvegicus*	RGSC6.0(rn6)	UCSC Genome Browser
*Bos taurus*	bosTau7	UCSC Genome Browser
*Monodelphis domestica*	monDom5	UCSC Genome Browser
*Ornithorhynchus anatinus*	ornAna1	UCSC Genome Browser
*Xenopus tropicalis*	xenTro3	UCSC Genome Browser
*Danio rerio*	danRer7	UCSC Genome Browser
*Caenorhabditis elegans*	ce10	UCSC Genome Browser

### Identification of tRFs from sRNA-seq datasets

tRNA-derived RNA fragments(tRFs) are 14–32 nt single-stranded RNAs (25,26). They are produced from pre-tRNAs (3′U tRFs) or mature tRNAs (3′ CCA tRFs, 5′ tRFs, tRF-i) (25). mirDeep2 (27) was first used to annotate miRNA sequences from TCGA raw sequencing data of small RNAs and the unaligned short sequences were kept for further analysis. tRFfinder (28) was then applied to identify tRFs. We next classified and annotated the identified tRFs to tRF-5 (5′ tRFs), tRF-3(3′ CCA tRFs), tRF-1 (3′U tRFs) and tRF-novel (tRF-i). The annotated tRFs were deposited in deepBase v3.0

### Expression analysis of various kinds of RNAs

For RNA-seq data, after downloading and classification, the raw data were recomputed as the FPKM (Fragments Per Kilobase of transcript per Million mapped reads) values to calculate the expression of genes. The expression in different tissues or life stages were normalized by *z*-score or mean and deposited in deepBase v3.0.

For sRNA-seq data, miRNA data in RPM form were collected to determine the expression amounts. snoRNA annotations were downloaded from snoDB. We counted the snoRNAs with featureCounts (parameters: -M -s1 -fraction).

For exosome data, based on a series of alignment results and a description file downloaded from ERCC, the expression levels of mRNAs and lncRNAs were integrated in RPKM format, and those of miRNAs and circRNAs were collected in RPM format.

### Differential expression analysis

The gene expression profiles identified from RNA-seq data were reannotated to GRCh38 and were then separated into different subclasses of RNAs. EdgeR (29) was used to perform differential expression analysis between cancer and normal samples. The differential expression of lncRNAs, mRNAs, small RNAs, tRNAs and snoRNAs was analyzed separately and then a comprehensive and detailed expression variation network of cancer RNAs was constructed. The differential expression changes and FDR values were deposited in deepBase and displayed in the web page.

### Predicting functions of ncRNAs from RNA–RNA coexpression networks

With a considerable amount of data, we developed a pipeline to predict functions of ncRNAs from RNA–RNA coexpression networks. Given the distribution of RNA expression values cannot be treated as normal, spearman correlation coefficient has better performance than pearson correlation coefficient (30). So, the expression correlations between ncRNAs and protein-coding genes were estimated using spearman correlation analysis in the *R* stats package and the *P*-values were adjusted with the FDR (False Discovery Rate) (31). Protein-coding genes with correlation values higher than 0.5 and *P*-values ≤0.05 were considered as coexpressed genes. GO and KEGG analyses were completed by the R clusterProfiler package (32).

### Prognostic analysis of differentially expressed ncRNAs

LncRNAs with apparent changes (|log FC| ≥ 1, *P*-value < 0.05) were collected from the differential expression data and clinical data from TCGA to complete a survival analysis using univariate Cox regression. Once an RNA was identified as apparently differentially expressed in one kind of cancer, it was brought into the analysis. deepBase set the log-rank *P*-value ≤0.05 to determine whether an RNA had an influence on patient survival. A KM survival plot was drawn for the qualified lncRNAs (33).

## DATABASE CONTENT AND WEB INTERFACE

### Web-based exploration of sRNAs, lncRNAs, circRNAs and tRFs

**Figure 2.**
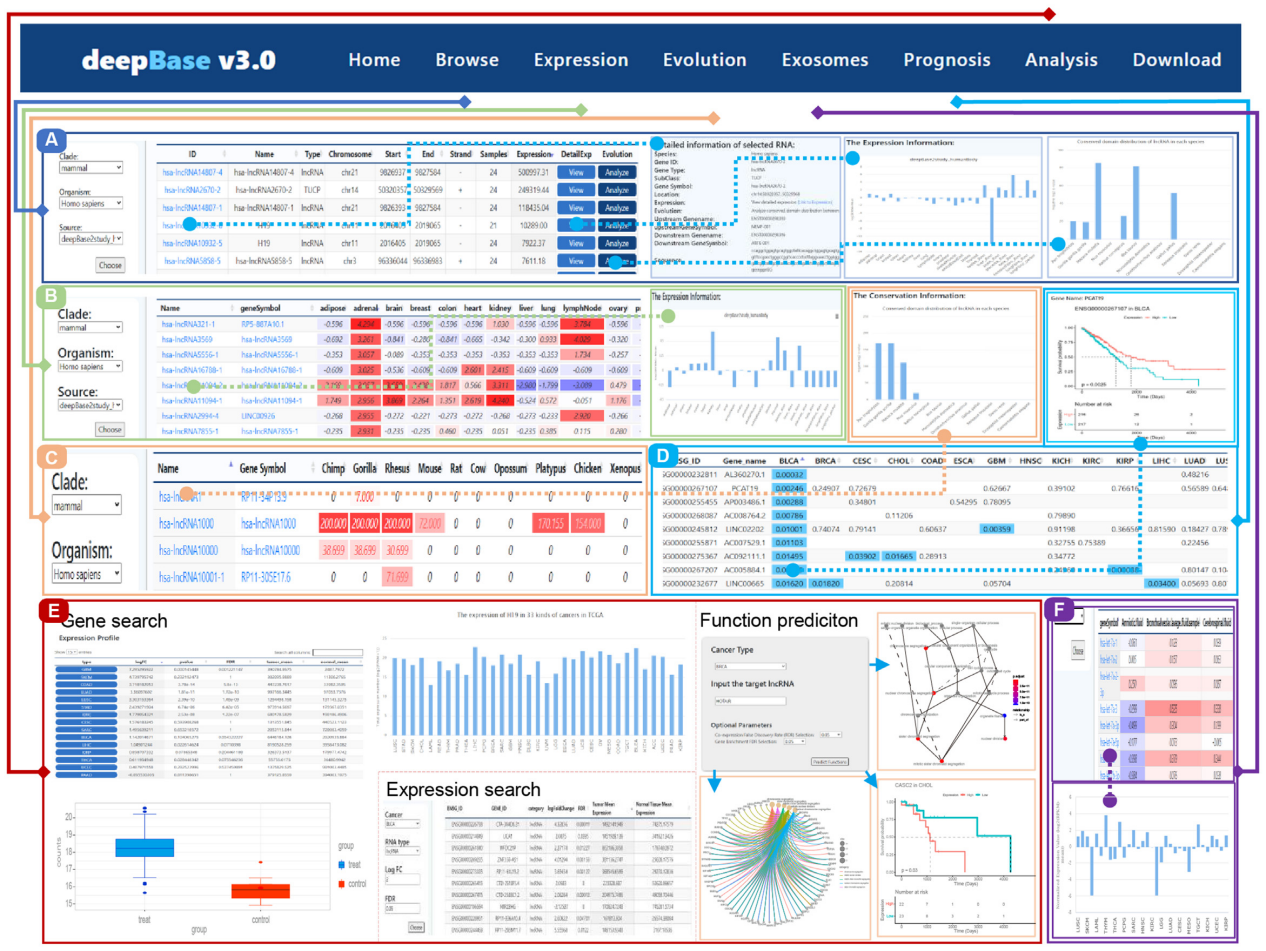
Introduction and usage of deepBase v3.0. (**A**) Browse page for ncRNAs with its detailed information, expression, and evolutionary conservation pages. (**B**) Expression page for ncRNAs with a detailed expression profile. (**C**) Evolutionary conservation of lncRNAs across 14 species. (**D**) Prognoses associated with cancer-related lncRNAs and detailed pages containing KM-plots of corresponding ncRNAs. (**E**) Multiple analysis interfaces for gene, expression, and function predictions. (**F**) Exosome expression page and detailed information for exosome expression profiles.

deepBase provides genome-wide identification of multiple types of RNAs, from lncRNAs to different types of small RNAs. In the Browse section, there are four web pages for user to browse different kinds of RNAs with annotations and expression profiles (Figure 2).

The Browse pages for lncRNAs, small RNAs and circRNAs display identified and reannotated RNAs from different experiments with their detailed information including the genomic locus, strand, length, type, total expression and expressed sample numbers. Users can sort the data table by the ‘Sample’ column to determine the universality of RNA expression. Some RNAs are widely expressed in all samples, while some are expressed in only a few samples. By sorting the data table by the ‘Expression’ column, users can obtain the expression conditions of RNAs. On the lncRNAs and smallRNAs page, we provide an outbound link to a new page showing the detailed expression in different samples or tissues. User can simply click the gene name to obtain detailed information.

deepBase provides an overview of tRFs identified from TCGA deep-sequencing data and shows the type, expressed sample number and expression details in multiple cancers. The naming rules are similar to those we used to name the lncRNAs in deepBase v2.0. Users can also click the tRF name to see information about its tRNA location, sequence and structure. An outbound link to RNAfold for the 2D structure is also provided.

### Expression profiles of various ncRNAs

Quantification of ncRNAs expression is one of the most important features of studies on RNA, and specific expression in certain stages, tissues or cells can imply the functions of ncRNAs in biological processes (34).

The expression sections are divided into two parts: normal tissues and cancers. In the normal tissue part, deepBase provides the expression profiles of all mRNAs, lncRNAs, miRNAs, circRNAs and small ncRNAs. deepBase v3.0 shows the normalized expression values (normalized by *z*-score or mean value) of RNAs in the form of a heatmap to give users a clear view of the relative expression in different tissues or samples. Users can click the gene name and jump to a detailed expression page. In the cancer part, the same normalization is applied to samples from different cancer types to show the expression differences between cancers. TCGA and ICGC data are displayed separately. The cancer small RNA page data are RNA-seq data, while the miRNA page data are sRNA-seq data, and the two types of data change in parallel. On the basis of the re-analysis of sRNA-seq data from TCGA, deepBase v3.0 also displayed the expression profiles of snoRNAs in various cancer types.

### Expression profiles of exosomes

Extracellular RNAs (exRNAs) are a type of RNA molecule that is present in various biological fluids. ExRNAs from heterogeneous populations including small RNAs, circRNAs, lncRNAs and mRNAs. They exist in free form or associate with proteins to form complexes, participating in a variety of cell-to-cell communications and play significant roles in cancer and other diseases. More than 2500 sample data downloaded from the ERCC database were analyzed to construct an expression map for different exosomes and to provide an overview of human exosomes RNA expression. Users can obtain direct views of exRNA expression in different biofluids and of the expression specificity of exRNAs.

### Prognostic analysis

With RNA-seq and sRNA-seq sequencing data, we also collected clinical data from TCGA and ICGC for deepBase v3.0. Combining expression data and clinical data, we applied univariate Cox regression to all differentially expressed genes (|log FC| ≥ 1, *P*-value < 0.05). deepBase shows all survival log-rank *P*-values in a data table. Genes that are not expressed in a specific cancer type or show no relationship to survival are not displayed. KM survival curve plots are provided for survival-related RNAs that pass the log-rank *P*-value threshold of 0.05.

### Interactive analysis for different kinds of ncRNAs

deepBase provides several analysis interfaces for users to take in-depth looks at different kinds of RNA in different features.

The gene search page shows the detailed expression of a single RNA in different types of cancer. This page consists of four parts. The left search bar includes the input box and guide. In the right section, a data table shows the expression and related information, and a boxplot shows the specific expression in one cancer type. Users can click on the cancer name in the data table to change it. A bar plot shows the cancer-wide expression. This page was designed to give users a direct and quick access to specific RNA information.

The expression page displays an expression matrix of different types of RNA in a single cancer type, and users can set the *P*-value and FDR cutoff to obtain a custom data table for further inspection. The search and expression pages provide user with quick and easy ways to obtain primary information from cancer-related RNA studies.

A web-based tool to predict ncRNA functions in cancer was developed based on coding and non-coding coexpression networks. There are four parameters to customize: the cancer type, ncRNA ID or gene name, co-expression FDR and enrichment analysis FDR. After submitting data, a user jumps to a new result page that displays the GO enrichment results, the KEGG enrichment results and a Kaplan–Meier plot. While only the biological process (BP) GO terms are shown on the webpage, a text file containing all three kinds of GO terms can be downloaded for further study. The user can download the specific plot or the zipped data of all plots and data tables.

## CONCLUSIONS

We introduce deepBase v3.0, which has significantly better web modules and functionalities than deepBase v2.0 (22). Previous versions of deepBase (22) have focused mainly on the expression patterns of miRNAs, lncRNAs and circRNAs in normal tissues or cell lines. In comparison to the previous release, deepBase v3.0 has several advances and improvements in data features and functionality (Table 1): (i) deepBase v3.0 provides the most comprehensive expression analysis of sRNAs and lncRNAs by mining 67 619 datasets for 14 species from large consortium projects and public deep-sequencing data. This will generate numerous differentially expressed ncRNAs for functional studies by bench biologists. (ii) To the best of our knowledge, this is the first attempt to construct the expression patterns of tRFs and snoRNAs from thousands of cancer and normal samples. It may help biologists select disease-related tRFs and snoRNAs for further functional validation. (iii) Gene expression data for miRNAs, lncRNAs and circRNAs from 2549 exRNA sequencing datasets have been newly added to deepBase. These data will help biologists discover noninvasive ncRNA biomarkers. (iv) The novel ‘Prognosis’ module has been developed to illustrate the correlations between ncRNAs and patient survival by linking a large number of expression profiles of ncRNAs with clinical data. (v) A new ‘Analysis’ module allows researchers to deeply investigate the functions of lncRNAs and other ncRNAs in tumor tumorigenesis by performing differential expression analysis and functional prediction based on protein–lncRNA coexpression networks across 42 types of cancers.

## FUTURE DIRECTIONS

Various high-throughput sequencing methods, such as CLIP-seq, ChIP-seq and ribo-seq, have been developed to explore the biological function, regulatory networks and translational potential of ncRNAs. We are considering adding these kinds of data in the next version of deepBase to facilitate analyses and explorations of the complex regulation, functions and mechanisms of various types of ncRNAs. Moreover, more annotation data and additional species will be integrated to further expand this database. We will continue to improve the database to accept and analyze new data uploaded by users.

## DATA AVAILABILITY

deepBase v3.0 is freely available at http://rna.sysu.edu.cn/deepbase3/index.html. The deepBase data files can be downloaded and used in accordance with the GNU Public License and the licenses of primary data sources.
